# Migration in times of pandemic: SARS-CoV-2 infection among the Warao indigenous refugees in Belém, Pará, Amazonia, Brazil

**DOI:** 10.1186/s12889-021-11696-7

**Published:** 2021-09-13

**Authors:** Hilton Pereira da Silva, Isabella Nogueira Abreu, Carlos Neandro Cordeiro Lima, Aline Cecy Rocha de Lima, Alexandre do Nascimento Barbosa, Lehi Rodrigues de Oliveira, Mayumi Aragão Fujishima, Sandra Souza Lima, Vitor Nina de Lima, Socorro Castelo-Branco, Antonio Carlos Rosário Vallinoto

**Affiliations:** 1 Laboratório de Virologia, Instituto de Ciências Biológicas, Universidade Federal do Pará, Belem, Brazil; 2 Secretaria de Saúde do Município de Belém (SESMA), Belem, Brazil; 3 Faculdade de Medicina, Instituto de Ciências da Saúde, Universidade Federal do Pará, Belem, Brazil; 4 Programa de Pós-Graduação Saúde, Ambiente e Sociedade da Amazônia e Programa de Pós-Graduação em Antropologia, Universidade Federal do Pará, Belem, Brazil; 5 Laboratório de Estudos Bioantropológicos em Saúde e Meio Ambiente, Instituto de Filosofia e Ciências Humanas, Universidade Federal do Pará, Belem, Brazil

**Keywords:** SARS-CoV-2, Indigenous, Warao, Brazil, Migration

## Abstract

**Background:**

The emergence of the new causative agent of Severe Acute Respiratory Syndrome Coronavirus 2 (SARS-CoV-2) in the city of Wuhan, China, in December 2019, and its spread worldwide, led the World Health Organization (WHO) to declare a pandemic. The disease has caused high mortality among traditional populations and the most socially vulnerable groups such indigenous and refugees. The present study aims to investigate the prevalence of anti-SARS-CoV-2 IgG antibodies in the population of Venezuelan indigenous Warao refugees residing in private and public shelters in the city of Belem, capital of Para State, in the Brazilian Amazon.

**Methods:**

One hundred one individuals of both sexes (43 men and 58 women) with ages varying from 18 to 77 years (average of 36 years) were investigated. Whole blood samples were collected and subsequently separated into plasma and leukocytes. Serological analysis was performed using an enzyme-linked immunosorbent assay - ELISA (Anti-SARS-COV-2 S1 IgG, EUROIMMUN, USA).

**Results:**

The results indicate a positive serum prevalence of 83.2% (84), of which 77.6% (45/58) were females and 90.7% (39/43) were males. An indeterminate profile was observed in 6.9% (7), where it was not possible to confirm the presence of antibodies, and 9.9% (10) individuals were negative for IgG antibodies.

**Conclusions:**

The finding of the high seroprevalence of IgG anti-SARS-CoV-2 antibodies reveals a high exposure of the Warao population in Belem to infection with the new coronavirus. These results underscore the importance of maintaining epidemiological surveillance with testing in traditional populations due to the high possibility of spreading the virus, especially among the most socioeconomically vulnerable groups, which depend exclusively on the Unified Health System (SUS), such as refugees and indigenous people.

**Supplementary Information:**

The online version contains supplementary material available at 10.1186/s12889-021-11696-7.

## Background

The identification, in Wuhan, China, of a new virus responsible for a type of severe acute respiratory syndrome (Severe Acute Respiratory Syndrome, Coronavirus 2 - SARS-CoV-2) isolated for the first time, in December 2019, generated a worldwide alert [1]. Its transmission spread rapidly across the planet, leading to the declaration of a pandemic state by the World Health Organization (WHO) in January 2020 [2]. The transmission of SARS-CoV-2 occurs mainly through respiratory droplets, aerosol, direct contact with contaminated surfaces, and less often by the fecal-oral route [3]. The disease has disproportionally affected traditional populations and the most socially and economically vulnerable groups in Brazil and other countries [4, 5].

As of April 1, 2021, 12,658,109 cases and 317,646 deaths were confirmed in Brazil. In the state of Pará, the number of cases reached 420,372 cases and 10,503 deaths [2, 6]. Due to the growing number of cases, the collapse of the health system in several states, and the concern with the most vulnerable populations, such as indigenous peoples, quilombola groups (traditional communities formed by descendants of runaway slaves in Brazil), and refugees, the continuous seroepidemiological surveillance of SARS-CoV-2 is extremely important, since the spread of the virus can happen more quickly, and be more lethal among them [7–9].

The Warao are a native people of Venezuela, who originally lived in the delta of the Orinoco River. They are the second largest ethnic group in the country composing 4.39% of the country’s population. They live in family groups, and traditionally their main forms of subsistence were fishing, collecting of forest products, and selling of handicrafts [10, 11]. The immigration of the Warao to Brazil began in 2014, first to the State of Roraima, having grown rapidly since 2016, with the increasing deterioration of the economic and political situation in their country. From the capital, Boa Vista, they went by boat towards Manaus and from there they reached Belém and other cities in Pará and Maranhão states [12]. The immigration of the Warao has increased over the years, reaching an estimated 3000 in the Brazilian territory in search of better living conditions, employment, education and health [13].

In Brazil, despite their official refugee situation, the Warao have faced situations of extreme social and economic vulnerability in the urban areas which, associated with the suffering caused by the migratory process, generates high rates of morbidity and mortality due to infectious and chronic diseases, and reduced quality of life. They often live in the periphery of the cities, in precarious housing conditions, without access to environmental sanitation or treated water [14]. The situation of vulnerability in the new urban environment is aggravated by the presence of several co-infections acquired along their lives, such as the Human Immunodeficiency Virus (HIV), the Human T-lymphotropic Virus (HTLV), malaria, tuberculosis, syphilis, and diseases such as diabetes, obesity and malnutrition, all of which are found in high frequency among this population [8, 15, 16].

Studies of the epidemiological situation of the Warao in the Amazon are still limited [12, 16]. Like other traditional groups, they are exposed to a high risk of infection and, in Belem, along the year of 2020, there were several deaths potentially associated with COVID-19 among them. As a result of these factors, the present study sought to investigate the prevalence of anti-SARS-CoV-2 IgG antibodies in this migrant population and the potential implications for the group’s epidemiological profile.

## Methods

### Study population

The study was carried out from September 2020 to January 2021 in three different places of residence of the Warao in Belém, capital of the state of Pará, one coordinated by the municipality and two managed by the indigenous themselves where most of the population of the city resided.

After the explanation of the project’s objectives and the approval of its development by the leaders and the families, all the adults were invited to participate. The objectives and methodology of the study were explained to each participant with the help of interpreters in Spanish and Warao. All measures of safety and social distancing were kept to protect both the researchers and the participants from COVID-19 infection.

All individuals aged 18 years or older, who agreed to participate voluntarily in the study, answered a questionnaire (Supplementary file) containing questions regarding gender, risk factors for COVID-19 (hypertension, smoking, alcoholism, diabetes, asthma or bronchitis, cancer, cardiovascular disease, heart disease, sickle cell anemia, tuberculosis, autoimmune disease, and other chronic illnesses), the presence of symptoms of COVID-19 (fever, headache, flu or cold, cough, sore throat, body pain, abdominal pain, diarrhea, nausea, vomiting, loss of smell, loss of taste, and shortness of breath), practice of self protection (distancing and isolation in the family and on the street, remained at home, care going to the pharmacy, to the supermarket, to work, to the bank, and use of mask in different circumstances), diagnosis, and treatment (“were you diagnosed with COVID-19?”, “did you have treatment for COVID-19?”, “did you use any drugs for COVID- 19?”, “did you use traditional medicine?”, “were you hospitalized?”, and “did you have any contact with someone diagnosed with COVID-19?”).

The sample collections were carried out after the completion of the questionnaire and formal acceptance of the participants. 4 ml of peripheral venous blood were collected in a tube containing EDTA as an anticoagulant. The samples were transported to the Virology Laboratory at UFPA, subjected to centrifugation at 8000 rpm for 15 min to separate plasma and cell mass, and then stored at − 20 °C until the time of analysis.

### Ethical aspects

The study was approved by the Research Ethics Committees of the Health Sciences Institute of the Federal University of Pará (CAAE: 31800720.1.0000.0018) and by the National Research in Ethics Commission (CAAE: 33470020.0.1001.0018). The leaders of the Warao people were consulted previously, expressed community interest, and signed an agreement for participation in the study. The research was explained, and a written informed consent was obtained from all participants. All methods were carried out in accordance with relevant guidelines and regulations.

### Serological diagnosis

The serological analysis was performed by means of enzyme-linked immunosorbent assay - ELISA, for qualitative detection of antibodies of the IgG class specific for the S1 subunit of the SARS-CoV-2 spike protein (Anti-SARS-COV-2 S1 (IgG), EUROIMMUM, USA), following the manufacturer’s protocol. All methods were carried out in accordance with relevant guidelines and regulations.

### Statistical analysis

The characteristics of the population were described using descriptive statistics (categorical variables were presented in frequencies and percentages and numerical variables in medians and quartiles). The Kolmogorov-Smirnov test was performed to assess normality and the F test to assess the homogeneities of variances of continuous variables. Fisher’s exact test and G test were used to assess the association of risk factors, prevention measures, distance and social isolation in relation to the SARS-CoV-2 serology result. Then, multiple logistic regression analysis was applied, based on the Backward method. The Mann-Whitney U test was used to identify whether there was a significant difference in age in relation to sex. Information that had an undetermined serological result (6.9%; 7) was excluded from statistical analysis. The significance level of 5% was adopted. All analyzes were performed using BioEstat version 5.3.

## Results

### Seroprevalence of anti-SARS-CoV-2 IgG

Of the 171 people living in the three main places of residence of the Warao in Belém, a total of 101 adult individuals agreed to participate in the study and were tested. A total of 84 (83.2%) were seropositive for the anti-SARS-CoV-2 S1 IgG antibody, of which 77.6% (45/58) were females and 90.7% (39/43) were males, with no statistically significant difference between the sexes (*p* = 0.1084). Ten individuals (9.9%) were seronegative. Seven (6.9%) samples presented an indeterminate profile, and it was not possible to confirm the presence of antibodies in them. There was no significant difference in age between the sexes (*p* = 0.345), the median age of the study participants was 34.0 (Q25 = 23.0; Q75 = 42.0) for females, and 35.5 (Q25 = 27.0; Q75 = 44.0) for males.

### Socioeconomic and demographic aspects of the Warao in Belém

Most participants self-declared indigenous (78.7%; 74). Regarding marital status, the majority reported “living together” (39.3%; 37). As for education, 42 (44.7%) declared having no formal education. Everyone in the community speak Warao and some, especially men, speak some Spanish and Portuguese. As for time of residence in Belém, 72 (76.6%) of the Warao have lived in the capital for at least 1 year, the others arrived more recently. They all live in family groups and not as tribal units, and have been moving around in several shelters of the capital. In Belém they make a living by begging on the streets (called “gathering”), selling handycrafts, and with the financial support of the government.

### Risk factors and symptoms of COVID-19

The occurrence of risk factors was more frequent among seropositive individuals than among those seronegative for IgG anti-SARS-CoV-2 antibodies, however none of the results were statistically significant (Table 1).

**Table 1:** Risk factors for SARS-CoV-2 related to the seroprevalence of IgG anti-SARS-CoV-2 antibodies

Risk factors	Total ***n*** = 94	%	Positive ***n*** = 84	%	Negative ***n*** = 10	%	OR	IC 95%	***p***-values
**Hypertension**									
**Yes**	9	9.6	9	10.7	0	0.0	3.71960E+ 08	0.0 - ^a^	0.9990
**No**	85	90.4	75	89.3	10	100.0	Ref.		
**Smoking**									
**Yes**	25	26.6	23	27.4	2	20.0	2.34	0.30–18.21	0.4180
**No**	69	73.4	61	72.6	8	80.0	Ref.		
**Alcohol abuse**									
**Yes**	35	37.2	31	36.9	4	40.0	0.78	0.15–4.20	0.7760
**No**	59	62.8	53	63.1	6	60.0	Ref.		
**Diabetes**									
**Yes**	3	3.2	2	2.4	1	10.0	0.20	0.01–3.28	0.259
**No**	91	96.8	82	97.6	9	90.0	Ref.		
**Asthma or bronchitis**									
**Yes**	1	1.1	1	1.2	0	0.0	6.54708E+ 08	0.0 - ^a^	1.0000
**No**	93	98.9	83	98.8	10	100.0	Ref.		
**Cancer**									
**Yes**	1	1.1	1	1.2	0	0.0	6.54708E+ 08	0.0 - ^a^	1.0000
**No**	93	98.9	83	98.8	10	100.0	Ref.		
**Cardiovascular disease**									
**Yes**	1	1.1	1	1.2	0	0.0	0.88	0.0 - ^a^	1.0000
**No**	93	98.9	83	98.8	10	100.0	Ref.		
**Heart disease**									
**Yes**	3	3.2	3	3.6	0	0.0	7.41501E+ 08	0.0 - ^a^	0.999
**No**	91	968	81	96.4	10	100.0	Ref.		
**Sickle cell anemia**									
**Yes**	0	0.0	0	0.0	0	0.0	–		–
**No**	94	100.0	84	100.0	10	100.0			
**Tuberculosis**									
**Yes**	3	3.2	3	3.6	0	0.0	2.06304E+ 08	0.0 - ^a^	1.0000
**No**	91	96.8	81	96.4	10	100.0	Ref.		
**Autoimmune disease**									
**Yes**	2	2.1	1	1.2	1	10.0	0.0	0.0 - ^a^	0.999
**No**	92	97.9	83	98.8	9	90.0	Ref.		
**Another chronic illness**									
**Yes**	4	4.3	3	3.6	1	10.0	1.43366E+ 08	0.0 - ^a^	0.999
**No**	90	95.7	81	96.4	9	90.0	Ref.		

^a^ number tends to infinity; OR: Odds ratio (adjusted)

The report of respiratory symptoms presented up to 15 days prior to the collection of the blood sample was related to the seroprevalence of IgG anti-SARS-CoV-2 antibodies (37/84 [44%]; *p* = 0.0452). Of the symptoms presented between June 2020 until the time of sample collection, the most common were headache 50% (47), followed by the flu-like illness or cold symptoms (41.5%; 39), fever (39.4%; 37), but there were no significant differences (Table 2). Multiple logistic regression analysis revealed no association between risk factors, symptoms, and seropositivity for IgG anti-SARS-CoV-2 (log-likelihood = − 29.295; *p* = 0.275).

**Table 2:** Symptoms presented between June 2020 until the time of the interview according to the seroprevalence of IgG anti-SARS-CoV-2 antibodies

Symptoms	Total ***n*** = 94	%	Positive ***n*** = 84	%	Negative ***n*** = 10	%	OR	IC 95%	***p***-values
**Fever**									
**Yes**	37	39.4	35	41.7	2	20.0	0.27	0.01–10.41	0.481
**No**	57	60.6	49	58.3	8	80.0	Ref.		
**Headache**									
**Yes**	47	50.0	44	52.4	3	30.0	2.02	0.10–42.23	0.650
**No**	47	50.0	40	47.6	7	70.0	Ref.		
**Flu-like illness or cold**									
**Yes**	39	41.5	37	44.0	2	20.0	33.57	0.05–23,959.63	0.295
**No**	55	58.5	47	56.0	8	80.0	Ref.		
**Cough**									
**Yes**	36	38.3	35	41.7	1	10.0	166.63	0.13–213,238.36	0.161
**No**	58	61.7	49	58.3	9	90.0	Ref.		
**Sore throat**									
**Yes**	32	34.0	31	36.9	1	10.0	0.07	0.0–140.39	0.487
**No**	62	66.0	53	63.1	9	90.0	Ref.		
**Body ache**									
**Yes**	30	31.9	29	34.5	1	10.0	14.51	0.20–1035.90	0.219
**No**	64	68.1	55	65.5	9	90.0	Ref.		
**Abdominal pain**									
**Yes**	24	25.5	23	27.4	1	10.0	1.06	0.02–73.97	0.979
**No**	70	74.5	61	72.6	9	90.0	Ref.		
**Diarrhea**									
**Yes**	19	20.2	18	21.4	1	10.0	0.21	0.01–7.04	0.386
**No**	75	79.8	66	78.6	9	90.0	Ref.		
**Nausea**									
**Yes**	8	8.5	7	8.3	1	10.0	0,12	0.01–2.78	0.187
**No**	86	91.5	77	91.7	9	90.0	Ref.		
**Vomiting**									
**Yes**	5	5.3	5	6.0	0	0.0	3.27323E+ 10	0.0 - ^a^	0.998
**No**	89	94.7	79	94.0	10	100.0	Ref.		
**Loss of smell**									
**Yes**	19	20.2	16	19.0	3	30.0	0.10	0.0–44.14	0.453
**No**	75	79.8	68	81.0	7	70.0	Ref.		
**Loss of taste**									
**Yes**	23	24.5	20	23.8	3	30.0	0.01	0.0–15.64	0.213
**No**	71	75.5	64	76.2	7	70.0	Ref.		
**Shortness of breath**									
**Yes**	28	29.8	25	29.8	3	30.0	5.82	0.04–776.80	0.481
**No**	66	70.2	59	70.2	7	70.0	Ref.		

^a^ number tends to infinity; OR: Odds ratio (adjusted)

### Distancing and social isolation

Of the 94 positive and negative participants for anti-SARS-CoV-2, 59.6% (56) reported having maintained social distancing and social isolation during the pandemic, limiting the exit from shelters to the minimum possible. Regarding the activity routine, 17 (16.0%) stated that they stayed at home since the beginning of the pandemic, 10.6% (10) went out occasionally to shop, 19.1% (18) went out every day to do some activity and 53.2% (50) said they left only for essential purchases, such as food, medicines and basic products. The frequency of departures per week was 34.0% (32) for once a week, 24.5% (23) for twice a week, and 34% (32) for three times a week or more. Of the participants, 88.3% (83) reported that only family members entered their shelter, while 10.7% (11) received some type of visit, such as family and friends (Table 3).

**Table 3:** Distance and social isolation measures carried out by the participants according to the seroprevalence of IgG anti-SARS-CoV-2 antibodies

Social activities	Total n = 94	%	Positive = 84	%	Negative = 10	%	OR	IC 95%	***p***-values
**Distance and isolation**									
**Yes**	56	59.6	51	60.7	5	50.0	1.52	0.36–6.31	0.566
**No**	38	40.4	33	39.3	5	50.0	Ref.		
**Stayed at home**									
**Yes**	85	90.4	75	89.3	10	100.0	0.0	0.0 - ^a^	0.999
**No**	9	9.6	9	10.7	0	0.0	Ref.		
**Going to the pharmacy**									
**Yes**	56	59.6	48	57.1	8	80.0	0.60	0.10–3.51	0.1943
**No**	38	40.4	36	42.9	2	20.0	Ref.		
**Going to the supermarket**									
**Yes**	74	78.7	65	77.4	9	90.0	1.03	0.11–10.10	0.4564
**No**	20	21.3	19	22.6	1	10.0	Ref.		
**Going to the work**									
**Yes**	23	24.5	19	22.6	4	40.0	0.82	0.16–4.18	0.808
**No**	71	75.5	65	77.4	6	60.0	Ref.		
**Going to the bank**									
**Yes**	37	39.4	31	36.9	6	60.0	0.59	0.12–2.91	0.5150
**No**	57	60.6	53	63.1	4	40.0	Ref.		

^a^ number tends to infinity; OR: Odds ratio (adjusted)

Regarding the SARS-CoV-2 prevention measures (Table 4), 97.9% (92) of the interviewees said they used masks made of cloth while maintaining social distance, 20.2% (19) said they always use mask, and 77.7% (73) only sometimes. When asked about the use of the mask when leaving home, 87.2% (82) reported using it always, 6.4% (6) used it sometimes, 3.2% (3) used it rarely, and 3.2% (3) did not use it at all. Regarding the practice of hand washing with soap 54.3% (51) reported washing their hands several times a day, 41.5% (39) washed only sometimes and 4.3% (4) rarely washed hands.

**Table 4:** Prevention measures related to the seroprevalence of IgG anti-SARS-CoV-2 antibodies

Prevention measures for SARS-CoV-2	Total ***n*** = 94	%	Positive ***n*** = 84	%	Negative ***n*** = 10	%	OR	IC 95%	***p***-values
**Wore a mask while distancing**									
**Yes**	92	97.9	82	97.6	10	100.0	7.63	0.0 - *	1.000
**No**	2	2.1	2	2.4	0	0.0	Ref.		
**How often do you wear a mask?**									
**Sometimes**	73	77.7	66	78.6	7	70.0	1.82	0.39–8.46	0.444
**Always**	19	20.2	16	19.0	3	30.0	Ref.		
**Never**	2	2.1	2	2.4	0	0.0	6.47232E+ 08	0.0 - ^a^	0.999
**Wore mask when on the street?**									
**Rarely**	3	3.2	3	3.6	0	0.0	76,472,871.59	0.0 - ^a^	0.999
**Sometimes**	6	6.4	6	7.1	0	0.0	3.60610E+ 08	0.0 - ^a^	0.999
**Always**	82	87.2	72	85.7	10	100.0	Ref.		
**Never**	3	3.2	3	3.6	0	0,0	7.57779E+ 08	0.0 - ^a^	0.999
**Do you wash your hands with soap?**									
**Only sometimes**	39	41.5	38	45.2	1	10.0	7.51	0.90–62.70	0.063
**Many times a day**	51	54.3	42	50.0	9	90.0	Ref.		
**Rarely**	4	4.3	4	4.8	0	0.0	1.76719E+ 08	0.0 - ^a^	0.999

^a^ number tends to infinity; OR: Odds ratio (adjusted)

## Discussion

The results show, for the first time in Brazil, the high level of exposure of the Warao population to infection by the SARS-CoV-2, demonstrated by a high prevalence of IgG antibodies, despite the fact that most individuals reported social distancing, wearing a cloth mask, and keeping isolation during the pandemic or even claiming to have been at home the entire time. However, in practice, it is commonly observed that Warao adults and children have been circulating on the streets of the capital during the period of the pandemic many times not wearing masks properly or even not using them at all. This gap between what was answered in the questionnaire and what was observed in the daily lives of the individuals, associated with the social habit of family visits, and collective housing in shelters without ideal sanitary conditions, may explain the high contact of this population with the virus, represented by the high prevalence of antibodies. We could also speculate that this discrepancy between what was reported and what was observed maybe due to some language bias during the interview, which could be a limitation of the study.

Furthermore, despite the guidance that the Warao population has been receiving from the municipal health agency on the importance of social distancing and the correct use of face mask, and hand washing, it is possible that their economic situation, living conditions and language barriers (difficulty in understanding Portuguese) may impact on their practice of preventive measures.

One of the characteristics of the Warao refugees in Brazil is their frequent displacement between states, cities, and between public and private shelters within the same city, which aggravates their contact with potential transmitters and also makes them potential carriers of the virus from one place to other. Historically, several vulnerability factors affect this migrant population in search of better quality of life in the country such as: sexual abuse, alcohol and drug use, food insecurity, survival in adverse street conditions, lack of access to health care, housing without basic sanitation, lack of access to drinking water, lack of jobs, in addition to discrimination and xenophobia [16].

The high prevalence of antibodies in the Warao population demonstrated in this study is similar to that found recently in the Xikrin of Bacajá ethnic group in the Xingu region, also in the State of Pará [9] in which 73% of the population also had anti-SARS-CoV-2 antibodies. The high prevalence among the migrants in Belem is possibly due to their daily activities in the streets searching for resources (called by them “gathering”), precarious housing, and the habit of family visiting among the shelters, which puts them in a situation of high risk of infection by the SARS-CoV-2, differently of the Xikrin of Bacajá population which live in a semi-isolated village, but relatively close to the local rural population [9, 17]. In addition, as mentioned above, the risk of infection likely increases as individuals demonstrate difficulties in following the recommendations for the use of mask and social distancing, associated with cultural factors, life in precarious sanitary conditions, and have low income as revealed by the responses to the epidemiological questionnaire.

The serological results highlight the vulnerability of the Warao, and the importance of maintaining epidemiological surveillance in traditional groups, since the virus has shown a high capacity for infection and community dissemination, especially among those segments of the population that are most socially and economically vulnerable and depend mostly of the public Unified Health System (*Sistema Único de Saúde - SUS*), as is the case of indigenous people, the refugees, and most of the poor and the black population of Brazil [7, 18].

The transmission of SARS-CoV-2 among indigenous populations is of great concern since they are among those with the lowest family income and poorer health in Brazil. In addition, traditional groups historically have difficulty accessing basic and specialized health care programs, depend exclusively of the SUS to assist all cases of COVID-19, and have shown a high rate of morbidity and mortality [7, 19]. In addition, there are cultural issues that hinder the use of masks and social isolation, as well as issues of access to water, housing conditions and the sharing of household items, which may impact the practice of non-pharmacological preventive measures [17, 20].

Although we have not observed seropositivity in tests performed with samples collected before the pandemic (data not shown), which minimizes the occurrence of cross-reaction, a possible limitation of our study in relation to seroprevalence values, could be the fact that the anti-SARS-CoV-2 S1 IgG antibody test used herein was not validated for the local population in which other infections are endemic and not prevalent in Europe or North America, where validation of the tests is commonly performed.

## Conclusions

Considering all the economic, cultural, social and environmental vulnerability factors faced by the indigenous immigrant population to Brazil, it is likely that the number of cases will increase as the pandemic becomes more virulent due to the new viral strains.

The results of in this study were sent in the form of a technical report to the public health department of the municipality of Belém, in order to support the public authorities with information on the scope of the pandemic among the Warao community, aiming for the promotion of interventions more appropriate to their socio-cultural context. We believe that the results presented here can serve as basis for understanding how refugee communities living in the same conditions as the Warao may be exposed to the spread of pathogens whose dissemination occurs by the same means as SARS-CoV-2.

Finally, the results presented here demonstrate the importance of continuous epidemiological surveillance, as well as the intensification of the vaccination campaign on traditional, migrant and refugee populations, in order to minimize the impact of the pandemic on these vulnerable communities.

## Supplementary Information

**Additional file 1.** COVID-19 Questionnaire.

## Data Availability

The datasets generated and/or analyzed during the current study are not publicly available to protect the privacy of participants, but are available from the corresponding author on reasonable request.
