# Supplementary material for: Migration in times of pandemic: SARS-CoV-2 infection among the Warao indigenous refugees in Belém, Pará, Amazonia, Brazil
Source: BMC Public Health. 2021 Sep 13;21:1659. doi: 10.1186/s12889-021-11696-7 (PMC8435358; doi:10.1186/s12889-021-11696-7)
Supplement: Supplementary file 1 — Additional file 1. COVID-19 Questionnaire. [file 12889_2021_11696_MOESM1_ESM.docx]

**COVID-19 QUESTIONNAIRE**

Data: ____/____/_____ (# ID): ________________

Population: _______________________

Name: _________________________________________________ Sex: ______ Date of birth: ____/_____/____ Age_________

Blood type: ( ) A+ ( ) A- ( ) AB+ ( ) AB- ( ) B+ ( ) B- ( ) O+ ( ) O- ( ) unknow

1. Where were you born (city/state)? ____________________
2. In which municipality do you reside? _____________________
3. How long have you lived in this location? ( ) < 1 year ( ) 1 - 3 years ( ) 4 - 7 years ( ) > 7 years
4. Regarding your skin color, how do you rate yourself? ( ) White ( ) Black ( ) Brown ( ) Yellow – Asiatic/Indigenous
5. What is your marital status? ( ) Married or "live together" ( ) Single ( ) Divorced ( ) Widower
6. Education level: ( ) Incomplete elementary school ( ) Complete elementary school ( ) Incomplete high school ( ) complete high school ( ) Incomplete higher education ( ) Complete higher education ( ) incomplete postgraduate ( ) Complete postgraduate ( ) Illiterate
7. Family income (minimum wages): ( ) < 1 ( ) 1 ( ) 2 ( ) 3 ( ) 4 ( ) 5 ( ) > 5
8. Do you or your family receive government aid? ( ) Yes ( ) No
9. Do you do any health monitoring? ( ) No ( ) Yes
10. If yes, when was the last time? ( ) One month ago ( ) 6 months ago ( ) > 6 months
11. If yes, where? ( ) Public health service- SUS ( ) Private health service
12. Do you smoke cigarettes? ( ) Yes ( ) No
13. If yes, how many cigarettes per day in the last week? ( ) Less than 10 ( ) Between 10-20 ( ) +20
14. Do you drink alcoholic beverages? ( ) Yes ( ) No ( ) Sometimes
15. If yes, how many times a week? ( ) 1 - 3 ( ) 4 - 6 ( ) Every day
16. Have you been vaccinated with BCG?? ( ) Yes ( ) No
17. Have you been vaccinated with H1N1? ( ) Yes ( ) No
18. Are you part of one of these risk groups for COVID-19? ( ) Hypertension ( ) Diabetic ( ) Asthmatic ( ) Autoimmune disease ( ) Cardiovascular disease ( ) Tuberculosis ( ) Sickle cell disease ( ) Obesity ( ) Cancer ( ) None
19. Did you have flu/cold symptoms in the last 15 days? ( ) Yes ( ) No
20. Clinical signs and symptoms of respiratory infection from January 2020 to the present:

Fever: ( ) Yes ( ) No _____^o.^C ( ) Not Measured

Headache: ( ) Yes ( ) No

Coryza: ( ) Yes ( ) No

Cough: ( ) Yes ( ) No Cough Characteristics: ( ) dry ( ) with secretion

Sore throat: ( ) Yes ( ) No

Body pain: ( ) Yes ( ) No

Abdominal pain: ( ) Yes ( ) No

Diarrhea: ( ) Yes ( ) No

Nausea: ( ) Yes ( ) No

Vomiting: ( ) Yes ( ) No

Loss of smell: ( ) Yes ( ) No

Loss of taste: ( ) Yes ( ) No

Shortness of breath: ( ) Yes ( ) No

Fatigue: ( ) Yes ( ) No

21) Did you have contact with someone diagnosed with Covid-19? ( ) Yes ( ) No

22) Did you make social distancing/isolation during the Covid-19 epidemic? ( ) Yes ( ) No

23) Have you traveled during the epidemic (since January 2020)? ( ) Yes ( ) No

24) In what period? ( ) Before the epidemic ( ) Beginning of the epidemic ( ) During the epidemic

25) What was the destination? Brazil ( ) Locals:____________________________ Abroad ( ) Local:_______________

26) Did you go out during social distancing? ( ) Yes ( ) No

27) If yes, for what reason? ( ) pharmacy ( ) supermarket ( ) bank ( ) work ( ) others

28) How often per week? ( ) One time ( ) Twice ( ) Three or more

29) Did you wear mask when leaving the distance? ( ) Yes ( ) No

30) How often? ( ) Always ( ) Sometimes ( ) Never

31) Do you have a health insurance? ( ) Yes ( ) No

32) Have you been diagnosed with Covid-19? ( ) Yes ( ) No

33) If yes, which method? ( ) RT-PCR ( ) Serological test ( ) RT-PCR + Tomography ( ) Serology + Tomography

34) Did you get service? ( ) Yes ( ) No

35) If so, where did you receive care? ( ) At home ( ) Emergency care unit ( ) Field hospital ( ) Public hospital - SUS

( ) Private hospital

36) Did you undergo treatment? ( ) Yes ( ) No

37) If yes, where? ( ) At home ( ) Emergency care unit ( ) Field hospital ( ) Public hospital - SUS

( ) Private hospita

38) Did you use medication? ( ) Yes ( ) No

39) If yes, which one? ( ) Hydroxychloroquine ( ) Ivermectin ( ) Nitazoxanide ( ) Anti-inflammatory ( ) Anticoagulant ( ) Antipyretic

( ) Azithromycin ( ) I don't know how to inform ( ) Other ( ) which?__________________________________________

40) Have you been hospitalized? ( ) Yes ( ) No

41) Did you need supplemental oxygenation? ( ) Yes ( ) No

42) Did you follow the recommendations to wash your hands with soap? ( ) Several times a day ( ) Only sometimes ( ) Rarely ( ) Never

43) Do you wear a mask when you're on the street? ( ) Sometimes ( ) Always ( ) Rarely ( ) Never

44) What type of mask? ( ) Homemade ( ) Surgical ( ) PFF2/N95 ( ) Other______________
